# Nutritional determinants of frailty in older adults: A systematic review

**DOI:** 10.1186/s12877-017-0496-2

**Published:** 2017-05-15

**Authors:** Laura Lorenzo-López, Ana Maseda, Carmen de Labra, Laura Regueiro-Folgueira, José L. Rodríguez-Villamil, José C. Millán-Calenti

**Affiliations:** 1 Universidade da Coruña, Gerontology Research Group, Instituto de Investigación Biomédica de A Coruña (INIBIC), Complexo Hospitalario Universitario de A Coruña (CHUAC), SERGAS, 15071 A Coruña, Spain; 2 Gerontology Research Group, Department of Biomedical Sciences, Medicine and Physiotherapy, Faculty of Health Sciences, Universidade da Coruña, Campus de Oza, 15071 A Coruña, Spain

**Keywords:** Nutritional status, Micronutrients, Macronutrients, Protein, Frail elderly

## Abstract

**Background:**

Frailty is a geriatric syndrome that affects multiple domains of human functioning. A variety of problems contributes to the development of this syndrome; poor nutritional status is an important determinant of this condition. The purpose of this systematic review was to examine recent evidence regarding the association between nutritional status and frailty syndrome in older adults.

**Methods:**

PubMed, Web of Science, and Scopus electronic databases were searched using specific key words, for observational papers that were published during the period from 2005 to February 2017 and that studied the association or relationship between nutritional status and frailty in older adults. The Preferred Reporting Items for Systematic Reviews and Meta-Analyses (PRISMA) Statement was followed to assess the quality of the included articles.

**Results:**

Of the 2042 studies found, nineteen met the inclusion criteria. Of these studies, five provided data on micronutrients and frailty, and reported that frailty syndrome is associated with low intakes of specific micronutrients. Five studies provided data on macronutrients and frailty, and among those studies, four revealed that a higher protein intake was associated with a lower risk of frailty. Three studies examined the relationship between diet quality and frailty, and showed that the quality of the diet is inversely associated with the risk of being frail. Two studies provided data on the antioxidant capacity of the diet and frailty, and reported that a high dietary antioxidant capacity is associated with a lower risk of developing frailty. Finally, seven studies evaluated the relationship between scores on both the Mini Nutritional Assessment (MNA) and the MNA-SF (Short Form) and frailty, and revealed an association between malnutrition and/or the risk of malnutrition and frailty.

**Conclusions:**

This systematic review confirms the importance of both quantitative (energy intake) and qualitative (nutrient quality) factors of nutrition in the development of frailty syndrome in older adults. However, more longitudinal studies on this topic are required to further understand the potential role of nutrition in the prevention, postponement, or even reversion of frailty syndrome.

**Electronic supplementary material:**

The online version of this article (doi:10.1186/s12877-017-0496-2) contains supplementary material, which is available to authorized users.

## Background

One of the populations with the most serious shifts in demographics is the world’s aging population. According to current estimations, the number of people aged 60 years or over is projected to grow from 901 million to 1.4 billion globally between 2015 and 2030 and will reach nearly 2.1 billion by 2050 [1]. The main reason that people are living longer is a reduction in mortality due to advances in health services, medicine, wealth and income, nutrition, behavior, and education [2]. However, living longer is not equivalent to being healthy, and increasing age is commonly related to different levels of frailty [3, 4].

The study of frailty has attracted enormous scientific interest in recent years because it affects multiple domains of human functioning, including gait, mobility, balance, muscle strength, motor processing, cognition, endurance, physical activity, and nutrition [5]. The decline across these multiple physiological systems triggers increased utilization of medical and social resources [3] with consequent economic expenditures.

Fried et al. [3] defined the presence of the frailty phenotype based on the presence of three or more of the following physical criteria: weight loss, exhaustion, physical activity, walking time, and grip strength. People are classified as frail if they meet three or more of these features, pre-frail if they meet one or two, and non-frail if they do not meet any of the criteria. Frailty is considered an early stage of disability, and as such it is characterized the potential for reversibility [6], meaning that appropriate interventions at the proper time can be used to prevent, postpone or even reverse this phenomenon [7].

A variety of problems contribute to the development of frailty and include, environmental factors such as physical activity [8] or a poorer nutritional status [9–11], both of which are important determinants in the development of frailty syndrome. Inadequate dietary intake has been associated with many conditions, such as an increased risk of chronic diseases [12], decreased antioxidant defenses [13], impaired immune responses [14], an increased risk of osteoporotic fractures [15], peripheral arterial disease [16], and frailty [10, 17]. Optimal nutrition is important for not only the prevention and treatment of different diseases [18], but also the facilitation of independence throughout the life cycle, which improves the quality of life of our elders, and ultimately promotes healthy aging [19]. In fact, the importance of nutrition as a means for postponing frailty in elderly people is a well-established phenomenon [20]. Nevertheless, a major obstacle to the success of nutritional interventions that is discussed in the literature has been the difficulty in comparing studies due to the use of multiple and different methods to measure nutritional status. To evaluate malnutrition, healthcare professionals and researchers rely on body mass index, anthropometry, biochemical markers, as well as a variety of nutritional screening tools [21]. Considering this difficulty and assuming that good nutritional interventions may play a role in the postponement or even reversion of frailty in the elderly, the aim of this systematic review was to critically appraise recent evidence pertaining to the association between nutritional status and frailty syndrome in older adults.

## Methods

### Data sources and search strategy

A systematic review of recent literature, published from January 2005 to February 2017 was performed. Three computerized electronic databases (PubMed, Web of Science, and Scopus) were searched using the following key search words: (“nutritional status” OR “nutrient deficiency” OR “nutrient deficiencies “OR “nutrient deficient” OR “nutrient intake” OR “nutritional intake” OR “food intake” OR “dietary intake” OR “dietary adequacy” OR “nutrition assessment” OR “nutritional assessment “OR “malnutrition” OR “undernutrition” OR “malnourishment”) AND (“frail” OR “frailty” OR “frail elderly”) AND (“elderly” OR “older adults” OR “older people” OR “geriatric”). All possible articles were merged into a single file, and duplicate records were removed after they were checked manually. Two independent reviewers evaluated the appropriateness of inclusion, and any conflicts that arose were discussed until a consensus was reached. In cases where a consensus was not reached, a third reviewer was included in the discussion.

### Inclusion and exclusion criteria

We included original scientific articles that met the following predefined criteria:

Setting: Community-dwelling or institutionalized frail elderly people who were aged 65 years or older were included. As a condition, frailty should be defined with a clear operational definition/measurement. The study was excluded if it defined frailty according to disabilities, comorbidities, nutritional status, or cognitive impairment. Outcomes: Only studies that examined the association or relationship between nutritional status and frailty as a primary outcome were included. Articles should have a record of micronutrient and/or macronutrient status and/or a clear operational definition/measurement of nutritional status; Language: Only full-text articles published in either English or Spanish were considered. It is important to note that we focused the search on undernutrition, malnutrition and nutritional deficiencies and that any research on overnutrition or obesity was not included.

Exclusion criteria: Abstracts, reviews, books, book chapters, letters, conference abstracts, short surveys, studies based on the description of a protocol, and interventional studies, as well as studies based on the perspective of the authors, and comments on an article were excluded.

### Data extraction

Studies were synthesized according to the following characteristics: authors and year, study design, country and sample characteristics (age and sex), setting, operational definition of frailty, nutritional measurement tools, prevalence/incidence of frailty and nutritional status, and main findings. This was a systematic review that did not require the ethics approval of an ethics committee. Because of the heterogeneity of the study designs, a narrative synthesis approach, rather than a meta-analysis, was utilized to examine the results. Different estimators of effect size (ES) were calculated according to the data to be compared. Cohen’s *d* values were reported as indicators of effect size (ES) for comparing the mean values. We interpreted the importance of the ES using the benchmarks for “small ES” (*d* = 0.2), “medium ES” (*d* = 0.5) and “large ES” (*d* = 0.8) as defined by Cohen [22]. The ES of the difference between two proportions was estimated according to the arcsine transformation by Cohen [22], and a Cohen’s *h* value was obtained. We interpreted the importance of these ES using the benchmarks for “small ES” (*h* = 0.2), “medium ES” (*h* = 0.5) and “large ES” (*h* = 0.8). Finally, odds ratios were converted into ES using a method proposed by Hasselblad & Hedges [23].

## Results

**Fig. 1:**
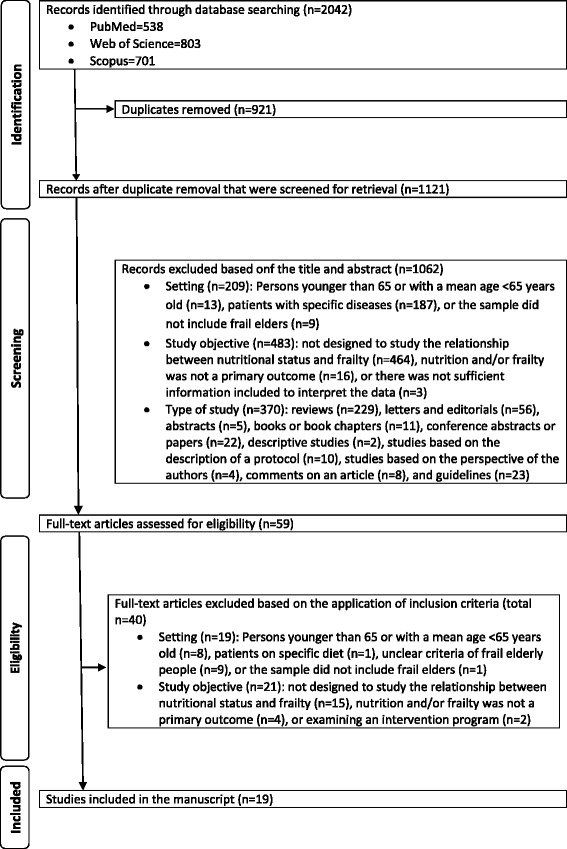
Flow diagram of study selection

The review procedure is described in Fig. 1. The Preferred Reporting Items for Systematic Reviews and Meta-Analyses (PRISMA) Statement was followed [24, 25] to assess the quality of the included articles (see Additional file 1). As shown in the figure, a total of 2042 studies were identified: After the removal of duplicates, 1121 were considered potentially relevant and were screened for pertinent content. From these studies, 1062 were excluded based on the title and abstract, while 59 were retrieved for full-text assessment. In the next phase, 40 articles were excluded based on the inclusion criteria (see Additional file 2): 19 were excluded for not meeting the setting characteristics, and 21 were excluded for not meeting the review objective. Ultimately, 19 studies met the criteria and were included in this review (see Figure 1).

### Participants and study characteristics

The included articles encompassed a sample of 22,270 older adults (63.2% women), with a mean age of 74.5 ± 7.0 years (extracted whenever possible). A total of 21,033 participants were community-dwelling elders, 111 lived in residential care facilities, and one study [26] recruited 1126 participants from both settings (community-dwelling people living either on their own or in a family house and those who were living in a nursing home). Eight studies were conducted in Europe [9, 26–32], seven in Asia [11, 33–38], and four in the United States of America [10, 17, 39, 40].

The identification of frail older people was based on the frailty phenotype [10, 17, 26–29, 33, 40] described by Fried et al. [3], on modifications of the frailty phenotype [9, 30, 32, 37, 38, 40], on the Study of Osteoporotic Fractures (SOF) Frailty Index [11, 33, 35, 36], and on the FRAIL scale [35]. Specific nutritional measurement tools employed in each paper are described in Table 1.

**Table 1:** Observational studies on nutrition and frailty (associations)

**Authors and Year**	**Study Design**	**Country and Sample Characteristics**	**Setting**	**Operational Definition of Frailty**	**Nutritional Measurement Tools**	**Incidence/Prevalence**	**Main Findings**
Bartali et al., 2006 [9]	Cross-sectional	Europe (Italy); *n* = 802; age, mean ± SD: 74.1 ± 6.5 years; 56% women	Community-dwelling participants aged 65 years or older who participated in the InCHIANTI study	Modified Fried frailty phenotype [3]: 4 of the 5 criteria of the Fried frailty phenotype (“weight loss” no included)	Dietary intake was assessed by a food-frequency questionnaire that was created for the EPIC study [47]. Data on food consumption were transformed into daily intake of energy, macronutrients, and micronutrients	20% frailty	Daily energy intake ≤21 kcal/kg and low intake of more than 3 nutrients were significantly and independently associated with frailty (OR 1.24, 95% CI: 1.02–1.5; OR 2.12, 95% CI: 1.29–3.50). Specifically, low energy intake of protein, vitamins D, E, and C and folate was related to frailty (OR 1.98, 95% CI 1.18–3.31; OR 2.35, 95% CI 1.48–3.73; OR 2.06, 95% CI 1.28–3.33; OR 2.15, 95% CI 1.34–3.45; and OR 1.84, 95% CI 1.14–2.98, respectively). Small Hasselblad & Hedges’ *d* ES, ranged from 0.05 to 0.2
Bollwein et al., 2013a [27]	Cross-sectional	Europe (Germany); *n* = 192; age, mean ± SD: 83.0 ± 4.0 years; 64.6% women	Community-dwelling older adults (>75 years)	Fried frailty phenotype [3]	Dietary quality was assessed by the alternate MDS of Fung et al. [48], who adapted the original score from Trichopoulou et al. [49]. MDS was calculated using a slightly modified version of the FFQ of the German part of the EPIC study (EPIC-FFQ) [50]	15.1% frailty and 41.1% pre-frailty	A healthy diet significantly decreased the risk of being frail (Q4 of the MDS, OR 0.26, 95% CI 0.07–0.98; Hasselblad & Hedges’ *d* ES 0.32)
Bollwein et al., 2013b [28]	Cross-sectional	Europe (Germany); *n* = 194; age, mean (range): 83.0 (75–96 years); 66.0% women	Community-dwelling older adults (>75 years)	Fried frailty phenotype [3]	Usual food intake was estimated using a slightly modified version of the FFQ of the German part of the EPIC study; (EPIC-FFQ) [50]	15.4% frailty and 40.5% pre-frailty	No significant differences were observed in the daily amount of protein intake between frailty groups. However, the distribution of protein intake was significantly different. Low morning protein intake values in frail, pre-frail and non-frail elders were 11.9, 14.9, and 17.4%, respectively). Small Cohen’s *h* ES, ranged from 0.05 to 0.14. Higher midday protein intake in frail, pre-frail and non-frail elders was 61.4, 60.8, and 55.3% respectively. Small Cohen’s *h* ES, ranged from 0.02 to 0.12
Bollwein et al., 2013c [29]	Cross-sectional	Europe (Germany); *n* = 206; age, mean (range): 83.0 (75–96 years); 66.0% women	Community-dwelling older adults aged 75 years or older	Fried frailty phenotype [3]	MNA® [41]	15.5% frailty and 39.8% pre-frailty; 15.1% at risk of malnutrition	A significant association between MNA total score and risk of frailty was found (2.2% of the non-frail, 12.2% of the pre-frail and 46.9% of the frail participants were at risk of malnutrition. Small Cohen’s *h =* 0.42 between non-frail and pre-frail but large ES between non-frail and pre-frail and frail (0.8 and 1.2, respectively). A significant association between 12 of the 18 MNA items and frailty was also observed
Boulos et al., 2016 [33]	Cross-sectional	Asia (Lebanon); *n* = 1200; age, mean ± SD: 75.7 ± 7.1 years; 42.3% women	Community-dwelling older adults (≥65 years) living in a rural setting	SOF index [51, 52]	MNA® [41]	36.4% frailty and 30.4% pre-frailty; 8.0% malnourishment and 29.1% at risk of malnutrition	Malnutrition and risk of malnutrition (i.e., poor nutritional status) were related to a significantly increased risk of frailty (OR 3.72, 95% CI 1.40–9.94; OR 3.66, 95% CI 2.32–5.76, respectively) but with small Hasselblad & Hedges’ *d* ES. Fourteen of the 18 MNA items were associated with frailty in age-adjusted analyses.
Chan et al., 2015 [34]	Longitudinal	Asia (China); *n* = 2724; age, mean ± SD: 71.8 ± 4.8 years; 50.3% women	Community-dwelling older adults (≥65 years)	FRAIL scale [53]	Assessment of baseline dietary intake through the FFQ. assessment of diet quality with the DQI-I [54]., and assessment of the adherence to the MDS using the revised method of Trichopoulou et al. [49]	1.1% frailty	Higher score of the “snacks-drinks milk products” significantly decreased the risk of being frail in a sex-age-adjusted model over a 4-year follow-up (adjusted OR 0.58, 95% CI 0.36–0.91). Better diet quality (higher DQI-I scores) significantly decreased the risk of being frail in both crude and sex-age-adjusted models over a 4-year follow-up (crude OR 0.61, 95% CI 0.43–0.86; adjusted OR 0.59, 95% CI 0.42–0.85, respectively). Nevertheless, no Hasselblad & Hedges’ *d* ES was observed. There was no association of MDS, “vegetables-fruits” pattern, or “meat-fish” pattern with incident frailty
Chang, 2017 [11]	Cross-sectional	Asia (Taiwan); *n* = 432; age, mean ± SD: 72.3 ± 10.0; 64.3% women	Community-dwelling older adults (≥65 years)	SOF index [51, 52]	MNA-SF® [41]	10.4% frailty and 23.6% pre-frailty; 30.6% at risk of malnutrition	Frailty was more prevalent in the group at risk of malnutrition. Frail status was a related risk factor at risk of malnutrition (OR = 8.78, with small Hasselblad & Hedges’ *d* ES, 0.32). Lower body mass index and lower skeletal mass indices were related to a higher risk of malnutrition. Frail people had a particularly high risk of malnutrition.
Chang & Lin, 2016 [35]	Cross-sectional	Asia (Taiwan); *n* = 152; age, mean ± SD: 80.8 ± 7.2 years; 18.4% women	Community-dwelling older adults (≥65 years)	SOF index [51, 52]	MNA® [41]	40.1% pre-frailty; 8.2% malnourishment and 34.9% at risk of malnutrition	The pre-frail group had a lower total MNA score than the non-frail group (β = −0.36, *p* < 0.001). Cohen’s *d* ES for mean scores was 0.75
El Zoghbi et al., 2014 [36]	Cross-sectional	Asia (Lebanon); *n* = 111; ≥65 years; 50.4% women	Institutionalized older adults	SOF index [51, 52]	MNA® [41]	37.9% frailty and 36.9% pre-frailty; 12.6% malnourishment and 48.7% at risk of malnutrition	The MNA score was inversely associated with the SOF Frailty Index (standardized beta coefficient − 0.18, 95% CI -1.46- -0.13). Mean scores comparison: small Cohen’s *d* ES (0.24) in malnutrition vs. risk of malnutrition, medium ES (0.65) in malnutrition vs. normal, and large ES (0.89) in risk of malnutrition vs. normal
Eyigor et al., 2015 [26]	Cross-sectional	Europe (Turkey); *n* = 1126; age range (65–85 years); 65.7% women	Community-dwelling older adults (living on their own or in a family house) and those living in nursing homes	Fried frailty phenotype [3]	MNA® [41]	39.2% frailty and 43.3% pre-frailty; 5% malnutrition and 27.5% at risk of malnutrition	Malnutrition increased the risk of frailty (OR 48.545, 95% CI 6.647–354.554). Large Hasselblad & Hedges’ *d* ES (0.89).
Jürschik et al., 2014 [30]	Longitudinal	Europe (Spain); *n* = 640; age, mean ± SD: 81.3 ± 5.0 years; 60.3% women	Community-dwelling older adults from the FRALLE survey	Slightly modified Fried frailty phenotype [3]: changes in metrics to characterize frailty [55]	MNA®, MNA-SF® [41]	9.6% frailty and 47% pre-frailty; 1.9% malnutrition and 19.8% at risk of malnutrition	Both the MNA (0.75, *p* < 0.001) and the MNA-SF (0.80, *p* < 0.001) were accurate in identifying frailty. Nevertheless, no Cohen’s *h* ES was observed
Kobayashi et al., 2013 [37]	Cross-sectional	Asia (Japan); *n* = 2108; age, mean ± SD: 74.7 ± 5.0 years; 100% women	Community-dwelling old women (≥65 years)	Modified Fried frailty phenotype [3]: includes only 4 components- slowness and weakness, exhaustion, low physical activity and unintentional weight loss [56]	Dietary protein intake source (animal or plant) and protein quality (amino acid components) were assessed by the BDHQ [57, 58]	22.8% frailty	Higher total, animal, and plant protein intake was inversely associated with frailty (adjusted OR for Q5 vs. Q1 0.66, 95% CI 0.46–0.96; 0.73, 95% CI 0.50–1.06; and 0.66, 95% CI 0.45–0.95, respectively). A higher intake of amino acids was associated with a lower prevalence of frailty (range of adjusted ORs for Q5 vs. Q1 0.67 for cysteine to 0.74 for valine). No Hasselblad & Hedges’ *d* ES were observed for any protein or amino acid intake
Kobayashi et al., 2014 [38]	Cross-sectional	Asia (Japan); *n* = 2121; age, mean ± SD: 74.7 ± 5.0 years; 100% women	Community-dwelling old women (≥65 years)	Modified Fried frailty phenotype [3]: includes only 4 components- slowness and weakness, exhaustion, low physical activity and unintentional weight loss [56]	Dietary TAC and food intake were assessed by the DHQ [58]	22.9% frailty	Higher intake of dietary TAC (FRAP, ORAC, TEAC, and TRAP assays) was inversely associated with frailty (adjusted OR for Q5 vs. Q1 0.35, 95% CI 0.24–0.53; 0.35, 95% CI 0.23–0.52; 0.40, 95% CI 0.27–0.60; and 0.41, 95% CI 0.28–0.62, respectively), with small Hasselblad & Hedges’ *d* ES. Higher food intake (coffee, vegetables, and fruit) was inversely associated with frailty (adjusted OR for Q5 vs. Q1 0.48, 95% CI 0.32–0.72; 0.47, 95% CI 0.33–0.69; and 0.71, 95% CI 0.49–1.03, respectively), without Hasselblad & Hedges’ *d* ES. Higher nutrient intake (vitamin A, α-carotene, β-carotene, β-carotene equivalent, cryptoxanthin, vitamin D, α-tocopherol, vitamin B6, folate, and vitamin C) was inversely associated with frailty (adjusted OR for Q5 vs. Q1 0.72, 95% CI 0.49–1.04; 0.68, 95% CI 0.47–0.98; 0.53, 95% CI 0.36–0.76; 0.47, 95% CI 0.33–0.66; 0.78, 95% CI 0.54–1.12; 0.67, 95% CI 0.46–0.98; 0.51, 95% CI 0.36–0.74; a 0.50, 95% CI 0.34–0.72; 0.52, 95% CI 0.36–0.76; and 0.61, 95% CI 0.42–0.88, respectively), without Hasselblad & Hedges’ *d* ES
Matteini et al., 2008 [39]	Cross-sectional	USA (Maryland); *n* = 703; age, range: 70–79 years; 100% women	Community-dwelling older women from the WHAS I and II	Fried frailty phenotype [3]	MMA, tHcy and cystathionine were assayed through stable isotope dilution capillary gas chromatography mass spectrometry with selected ion monitoring. Vitamin B6 was measured as pyridoxal 5-phosphate using high-performance liquid chromatography. Serum vitamin B12 and folate were measured by radiodilution assay	13.7% frailty	Increased concentrations of MMA (a marker of vitamin B12 deficiency) were related to greater odds of pre-frailty and frailty (OR 1.59, 95% CI 0.95–2.65, no Hasselblad & Hedges’ *d* ES; OR 2.33, 95% CI 1.14–4.77, small Hasselblad & Hedges’ *d* ES)
Michelon et al., 2006 [17]	Cross-sectional	USA (Maryland); *n* = 754; age, mean (range): 74.7 (70–80 years); 100% women	Community-dwelling older women from the WHAS I and II	Fried frailty phenotype [3]	Plasma carotenoids, retinol, and α-tocopherol were determined by high-performance liquid chromatography. Total carotenoids were calculated as the sum of α-carotene, β-carotene, β-cryptoxanthin, lutein/zeaxanthin, and lycopene (in μmoles/l). 25(OH)D was measured using a radioreceptor assay. Vitamin B6 status was assessed by pyridoxal 5-phosphate measurements using high-performance liquid chromatography. Serum vitamin B12 and folate were measured using RIA	11.4% frailty and 44.7% pre-frailty	Lower serum levels of total carotenoids, α-tocopherol, 25-hydroxyvitamin D, and vitamin B6 significantly increase the risk of becoming frail in age-adjusted regression models (age-adjusted OR for Q1 vs. Q2-Q3-Q4 2.50, 95% CI 1.51–4.14; 1.64, 95% CI 0.95–2.84, 1.71, 95% CI 1.00–2.94; and 1.79, 95% CI 0.99–3.24, respectively), with only small Hasselblad & Hedges’ *d* ES for lower serum levels of total carotenoids. Lower serum levels of β-carotene, lutein/zeaxanthin, and total carotenoids significantly increase the risk of becoming frail with advancing age, sociodemographic status, smoking status, and body mass index models (OR ranging from 1.82 to 2.45, *p* = 0.05), with small or no Hasselblad & Hedges’ *d* ES
Rabassa et al., 2015 [31]	Longitudinal	Europe (Italy); *n* = 769; age, mean ± SD: 72.7 ± 5.8 years; 55.4% women	Community-dwelling older adults from the Invecchiare in Chianti study	Fried frailty phenotype [3]	Habitual dietary resveratrol exposure was assessed. TDR was assessed through the Italian version of the FFQ developed and validated in the EPIC [47] and an ad hoc food-composition database on resveratrol [59, 60]. TUR was analyzed with the use of liquid chromatography-tandem mass spectrometry with a previous solid-phase extraction at baseline. TDR + TUD was measured and computed using Howe’s method [61]	4.4% frailty and 37.4% pre-frailty	TDR, TUR, and TDR + TUR concentrations were inversely associated with frailty risk over 3-years of follow-up but not after 6- and 9-years of follow-up (OR for comparison of extreme tertiles: 0.17, 95% CI 0.05–0.63; 0.32, 95% CI 0.09–1.11; and 0.11, 95% CI 0.03–0.45, respectively), with small and medium Hasselblad & Hedges’ *d* ES (0.42, 0.27 and 0.53, respectively)
Rahi et al., 2016 [32]	Cross-sectional	Europe (Paris); *n* = 1345; age, mean ± SD: 75.6 ± 5.1 years; 60.4% women	Community dwellers aged 65 years and above	Modified Fried frailty phenotype [3]: handgrip strength was replaced by the chair standing method; since walking speed was missed for many elders, the Rosow-Breslaw test was used [62]	Daily intakes of energy and protein were set at ≥30 kcal/kg body weight/d and ≥1 g/kg body weight/d,	4.1% frailty; 57.7% protein intake ≥1 g/kg body weight/d	A higher protein intake was associated with a lower prevalence of frailty because the association with a slow walking speed (OR = 0.63; with no Hasselblad & Hedges’ d ES
Semba et al., 2006 [10]	Longitudinal	USA (Maryland); *n* = 766; age, mean ± SD: 78.2 ± 7.6 years; 100% women	Community-dwelling older women from the WHAS-I	Fried frailty phenotype [3]	Nutrient concentrations were measured through blood analysis. Serum samples for total carotenoids, retinol, and α-tocopherol, levels were determined by high-performance liquid chromatography. Serum selenium and zinc levels were measured by graphite furnace atomic absorption spectrometry using a PerkinElmer AAnalyst 600 (Norwalk, CT) with Zeeman background correction. 25(OH)D was measured using a radioreceptor assay. Serum vitamin B12 and folate were measured using RIA	32.6% frailty	Lower levels of serum carotenoids and α-tocopherol significantly increase the risk of becoming frail over a period of 3 years (HR for Q1 vs. Q2-Q3-Q4 1.30, 95% CI 1.01–1.92; and 1.39, 95% CI 1.02–1.89, respectively). The number of nutrient deficiencies was associated with an increased risk of becoming frail over a period of 3 years (adjusted HR 1.10; 95% CI 1.01–1.209). No Hasselblad & Hedges’ *d* ES were observed.
Shikany et al., 2014 [40]	Longitudinal	USA; *n* = 5925; age, mean ± SD: 75.0 ± 5.7 years; 100% men	Community-dwelling from the Osteoporotic Fractures in Men (MrOS) study	Slightly modified Fried frailty phenotype [3]: due to the lack of data on body weight prior to enrollment, appendicular lean mass in the lowest quintile was used for the shrinkage component	Food intake was assessed through Block 98 of the FFQ [63, 64]. Diet quality was assessed with the DQI-R [65, 66]	8.4% frailty and 45.2% pre-frailty	At baseline: higher intake of fiber significantly decreased the risk of intermediate or frail status relative to a robust status (OR for Q5 vs. Q1 0.83, 95% CI 0.69–1.00; and 0.51; 95% CI 0.36–0.73, respectively). Higher intake of carbohydrate was significantly associated with reduced odds of frailty relative to a robust status (OR for Q5 vs. Q1 0.65; 95% CI 0.45–0.94). Higher intake of fat was significantly associated with greater odds of frailty relative to a robust status (OR for Q5 vs. Q1 1.61; 95% CI 1.12–2.31). DQI-R was inversely associated with frailty relative to a robust status (OR for Q5 vs. Q1 0.44, 95% CI 0.30–0.63). No Hasselblad & Hedges’ *d* ES were observed. Prospective analysis: DQI-R was inversely associated with the risk of intermediate or frailty status relative to a robust status (OR for Q5 vs. Q1 0.82, 95% CI 0.60–1.11; and 0.18, 95% CI 0.03–0.97, respectively) with no Hasselblad & Hedges’ *d* ES for the risk of frailty but medium *d* ES in frailty

*Abbreviations: BDHQ* Brief-type Diet History Questionnaire, *CI* Confidence interval, *DHQ* Diet History Questionnaire, *DQI-I* Diet Quality Index-International, *DQI-R* Diet Quality Index Revised, *EPIC* European Prospective Investigation into Cancer and Nutrition, *ES* Effect size, *FFQ* Food Frequency Questionnaire, *FRAIL* Fatigue, Resistance, Ambulation, Illness, Low Weight, *FRALLE* Frailty Lleida, *InCHIANTI* Invecchiare in Chianti, ageing in the Chianti area, *FRAP* Ferric Reducing Ability of Plasma, *MDS* Mediterranean-Diet Score, *MMA* Methylmalonic Acid, *MNA* Mini-Nutritional Assessment, *MNA-SF* Mini-Nutritional Assessment Short Form, *OR* Odds ratio, *ORAC* Oxygen Radical Absorbance Capacity, *RIA* Radioimmunoassay, *SOF* Study of Osteoporotic Fractures, *TAC* Total Antioxidant Capacity, *TDR* Total Dietary Resveratrol, *TEAC* Trolox Equivalent Antioxidant Capacity, *tHcy* Homocysteine, *TRAP* Total Radical-trapping Antioxidant Parameters, *TUR* Total Urinary Resveratrol, *WHAS* Women’s Health and Aging Study, *25(OH)D* Serum 25-hydroxyvitamin D

### Nutritional outcomes

#### Association between micronutrients and frailty

Five studies provided data on micronutrients and frailty [9, 10, 17, 38, 39] and used some specific micronutrients as a measure of nutrient intake. In all five studies, frailty syndrome was independently associated with a low intake of specific micronutrients. In a cross-sectional analysis of the InCHIANTI study, Bartali et al. [9] found that a low intake of certain micronutrients (vitamins D, E, and C, and folate) was significantly related to frailty independent of energy intake. In a cross-sectional multicenter study among three generations (dietetic students, their mothers, and their grandmothers), Kobayashi et al. [38] found that 10 of 12 micronutrients studied (vitamin A, α-carotene, β-carotene, β-carotene equivalent, cryptoxanthin, vitamin D, α-tocopherol, vitamin B6, folate, and vitamin C) were associated with a lower prevalence of frailty. By using a sample of community-dwelling women from the Women’s Health and Aging Studies (WHAS) I and II, Matteini et al. [39] showed that older women with increased concentrations of methylmalonic acid (MMA: a marker of vitamin B12 tissue deficiency) had 40%–60% greater odds of being pre-frail (*p*-values <0.07) and 1.66–2.33 times greater odds of being frail (*p*-values <0.02) compared to patients who were not frail. By using the same sample, Michelon et al. [17] reported that the age-adjusted odds ratios of being frail were higher for older women with lower levels of micronutrients: such as serum total carotenoids, α-tocopherol, 25-hydroxyvitamin D, and vitamin B6. Importantly, after adjusting for age, sociodemographic status, smoking status, and body mass index, the association between micronutrients and frailty was strongest for total carotenoids, β-carotene, and lutein/zeaxanthin. Finally, Semba et al. [10], also used data from the WHAS-I study, and showed that women in the lowest quartile of serum carotenoids and α-tocopherol had a significantly increased risk of becoming frail over a 3-year period. By applying a multivariate grouped-time Cox proportional hazards model, the number of nutrient deficiencies was also related to an increased risk of becoming frail.

#### The role of macronutrient intake in frailty

Five studies reported data on the relationships between macronutrients and frailty [9, 28, 32, 37, 40]. Three of the studies found that a higher protein intake was associated with a lower risk of frailty [9, 32, 37], whereas two studies reported that the amount of protein intake was not associated with frailty [28, 40]. In particular, Bartali et al. [9] found an association between low protein intake (lowest quintile; energy intake of ≤21 kcal/kg/day) and frailty after adjusting for energy intake. In a community-dwelling elder population from France, Rahi et al. [32] found that a 1 g/kg protein intake was associated with a lower prevalence of frailty, after adjusting for sociodemographic and clinical factors. In a multicenter cross-sectional study that explored the association between protein or amino acid intake and frailty, Kobayashi et al. [37] showed that a higher intake of total protein was significantly associated with a lower prevalence of frailty among older Japanese women, regardless of the protein source (animal sources: fish and shellfish, meat, eggs, and dairy products; plant sources: cereals, pulses, potatoes, confectionaries, fruits and vegetables), or the amino acid that composed the protein (leucine, isoleucine, valine, methionine, cysteine, branched chain amino acids, sulfur amino acids, and essential amino acids). Although amino acid consumption was inversely associated with frailty, the association of total protein intake was stronger than those of any individual amino acids. The authors concluded that neither the protein sources nor the type of amino acids were particularly relevant in the prevention of frailty. In another cross-sectional study that investigated the association between the amount and distribution of protein intake throughout the day (morning, noon, evening) and frailty in older German community-dwelling seniors, Bollwein et al. [28] found that the amount of protein intake was not associated with frailty or any of its individual criteria (the authors only found a significant trend concerning low physical activity). However, the distribution of protein intake throughout the day was significantly associated with frailty. Specifically, frail older adults showed a more uneven distribution of protein intake throughout the day with a lower morning intake and a higher midday intake than pre-frail and non-frail participants. In a longitudinal study that examined the association between baseline dietary variables and baseline frailty status, Shikany et al. [40] found that while a higher intake of fiber and carbohydrates significantly decreased the risk of intermediate or frail status relative to a robust status, a higher fat intake significantly increased the risk of being frail relative to a robust status. Notably, protein intake was not associated with the risk of frailty relative to a robust status [40].

#### Relationship between dietary patterns and frailty

One longitudinal study explored the relationship between dietary patterns and the four-year risk of frailty [34]. In a sample of Chinese community-dwelling older adults, Chan et al. [34] found that a higher score of “snacks-drinks-milk products” patterns decreased the risk of being frail. This association disappeared when the model was adjusted for age and sex, or for other demographic and lifestyle factors. No association with the incidence of frailty was found for “vegetables-fruits products” or “meat-fish products” patterns.

#### Relationship between diet quality and frailty

Three studies examined the relationship between diet quality and frailty [27, 34, 40], and showed that the overall quality of the diet was inversely associated with the risk of being frail. With the use of a Mediterranean-Diet Score (MDS) to evaluate a priori-defined dietary patterns, Bollwein et al. [27] found that compared to a less healthy diet, community-dwelling older adults who had the healthiest diet had a significantly decreased risk of being frail. The effect of the diet was graded, as manifested by the linear trend in the odds ratios (OR). Considering the singular frailty criteria, there was a significant and inverse association between “weight loss,” “low physical activity,” and “low walking speed” and the MDS. With the use of the Diet Quality Index-International (DQI-I) and the MDS, Chan et al. [34] explored the associations between dietary patterns and four-year incident frailty in older Chinese adults and found that participants with a higher DQI-I score (which represents a balanced diet in terms of energy and nutrient intake) had a reduced risk of frailty in both sex- and age-adjusted models. However, the authors did not find any association between MDS and frailty. Finally, in an analysis of the Osteoporotic Fractures in Men (MrOS) longitudinal study that used the Diet Quality Index Revised (DQI-R), Shikany et al. [40] found, that the DQI-R score in a cohort of older men was inversely associated with frailty status relative to a robust status at both a baseline and second clinic visit (a mean of 4.6 years later).

#### Antioxidant capacity of the diet and frailty

Two studies reported data on the antioxidant capacity of a diet compared to frailty [31, 38]. Both studies showed that a higher dietary antioxidant capacity is associated with frailty status. Specifically, in a cross-sectional multicenter study, Kobayashi et al. [38] found that a higher intake of dietary total antioxidant capacity (TAC) measured with four assays (ferric reducing ability of plasma (FRAP), oxygen radical absorbance capacity (ORAC), Trolox equivalent antioxidant capacity (TEAC), and total radical-trapping antioxidant parameters (TRAP)) was inversely associated with frailty. The intake of green tea, coffee, vegetables, and fruits which contributes to dietary TAC was also associated with lower odds of frailty, since the odds ratios were less pronounced than those for dietary TAC. Rabassa et al. [31] designed a longitudinal study that investigated the association of habitual dietary resveratrol exposure (measured by total dietary resveratrol (TDR), total urinary resveratrol (TUR), and the combination of both measures (TDR + TUR) with frailty syndrome and each of its 5 criteria at baseline and at 3-, 6-, and 9-year follow-up periods. The authors found that high habitual dietary resveratrol exposure was associated with a lower risk of developing frailty syndrome in older adults during the first 3 years of follow-up but not after 6- and 9-years follow-up periods, despite results that trended in the same direction. Considering individual frailty criteria during the 3-year follow-up period, and after adjusting for baseline frailty syndrome and for potential covariates (including energy intake), participants in the highest tertile of TDR + TUR had a lower risk of feeling exhausted than did those participants in the lowest tertile. No associations were observed for other frailty criteria. Considering TDR exposure, raw models identified a significant inverse association between this measure and low levels of physical activity at the 3, 6, and 9-year follow-up periods, although this relationship was not present in the adjusted models.

#### Relationships between the mini nutritional assessment (MNA®) and the mini nutritional assessment shot form (MNA-SF®) scores and frailty

Six studies evaluated the relationship between MNA and MNA-SF [41] scores and frailty [11, 26, 29, 30, 33, 35, 36], and revealed a significant association between malnutrition, the risk of malnutrition, and frailty status. Specifically, Chang [11] found that frail Taiwanese community-dwelling elders had a particularly high risk of malnutrition. Bollwein et al. [29] reported a significant association between frailty status and the three dimensions of the MNA score: MNA total scores, MNA-subscores, and 12 of the 18 MNA single items. Boulos et al. [33] reported a strong association between 14 of the 18 MNA items and frailty status. In addition, by applying a multivariate analysis, the authors found a strong and independent relationship between frailty and both malnutrition and the risk of malnutrition. In a pioneer study carried out in Asian pre-frail elders, Chang and Lin [35] revealed a relationship between pre-frail status and the total MNA score. Similarly, El Zoghbi et al. [36] showed that frailty was inversely correlated with the nutritional status of 111 institutionalized elders. In a multicenter study, Eyigor et al. [26] found that several socio-demographic factors, lifestyle variables, and clinical characteristics (such as malnutrition) were related to frailty. Specifically, the authors found that being malnourished increased the risk of being frail. Finally, in a sample of 640 community-dwelling Spanish elders, Jürschik et al. [30] found a significant association between the 5 frailty criteria and malnourishment, as identified by the MNA and the MNA-SF, and argued that both tests could be used to identify frail elders.

## Discussion

This systematic review regarding the relationship between nutrition measurements (micronutrients, macronutrients/protein intake, diet quality, antioxidant capacity, MNA or MNA-SF scores) and frailty in elderly people, provides current evidence of an association between many of these outcomes and frailty syndrome. The focus on malnutrition, at both the micro- and macronutrient levels is relevant because nutrition was identified in a recent systematic review as a means for delaying the onset of the negative consequences of frailty in older adults [20] as well as for the slowing the development and progression of frailty in elderly people [9]. Similar to a previous article [8], we required rigorous criteria to perform this review and to define frailty in older people; thus, trials that included the word “frail” in the title or in the abstract, and studies that did not rigorously define the word “frail”, were not included.

Five of the articles studied the association between micronutrients and frailty, and reported that low intake of specific micronutrients increased the risk of being frail [9, 10, 17, 38, 39]. Among the micronutrients that were studied, most of them had sequentially decreasing levels in non-frail, pre-frail, and frail older people. One important implication of the inverse association between micronutrients and frailty is that the intake of specific nutrients affect the health of older people and may lead to the development of frailty, among other important conditions. Indeed, different types of cancer are related to deficiencies of carotenoids and vitamins C and E [42]; cardiovascular disease is related to the impaired antioxidant capacity of vitamin E, β-carotene, and vitamin C [43], and sarcopenia is related to low serum concentrations of carotenoids and vitamin E [44]. A rich dietary intake of carotenoids and vitamins could be a potentially modifiable factor for preventing all these conditions. Therefore, it is important to teach our elders about foods that contain specific micronutrients. The goal could be to present specific dietary programs aimed at the avoidance of malnutrition while increasing the intake of foods that are rich in carotenoids and vitamins (such as vegetable foods) depending on the needs of each elder. Based on these findings, Bartali et al. [9] suggested that the quality of the diet, expressed by the intake of specific nutrients, should be included as part of frailty screening, diagnostic and treatment processes, because nutrition is a relevant factor that significantly affect the health of older adults.

Five studies [9, 27, 32, 36, 40] included in this review considered the role of macronutrient and protein intake in frail patients. Three of those studies [9, 32, 37] found that higher protein intake was associated with lower frailty risk, while only one study [28] found that it was actually the overall distribution of the protein throughout daily meals that was significantly associated with frailty. Specifically, Bollwein et al. [28] found greater uniformity in the pattern of protein intake in non-frail elders than in frail or pre-frail older adults. This result occurred with other findings and signaled the importance of ingesting a sufficient amount of protein with each meal, which is recommended at 25–30 g of high-quality protein per meal or approximately 1–1.2 g/kg per day [45].

In addition, the three studies that examined the relationship between overall diet quality and frailty [27, 34, 40] revealed that the quality of the diet is inversely associated with the risk of being frail, thus providing convergent evidence that a potentially modifiable factor, such as dietary intake, may play a crucial role in frailty status. As already suggested [27] and as proposed by the MDS, this effect could be mediated by a low intake of animal products and a high intake of fruits and vegetables. This result coincides with the previously mentioned micronutrient studies that indicated that the main nutritional sources of carotenoids and vitamin C were vegetable foods. Consistent with these results, this review found two studies [31, 38] that showed that a high intake of foods with high dietary antioxidant capacity, such as vegetables, fruits, coffee, and green tea, was associated with a lower risk of developing frailty. In summary, this and the previous findings indicate that a high-quality diet with satisfactory energy intake, the optimal intake of quality protein that is evenly distributed throughout all meals, and meals that are rich in antioxidants, are important factors for preventing and postponing the onset of frailty in older adults.

In is important to note that all the reviewed papers highlighted the importance of different nutritional factors in frailty, regardless of the type of study, the studied sample, or the instruments used to measure frailty and nutritional status.

Regarding the gap in the literature, future longitudinal studies with larger sample sizes and clinical trials are needed to further improve our knowledge regarding the associations between nutritional status and frailty. Specifically, future studies that examine the relationships between micro- and macronutrients concentrations and frailty are needed. It would also be interesting to determine whether different components of frailty are associated with the quality of the diet, independent of major confounders, in future studies.

This review has several limitations that should be mentioned. The main limitation is that most of the included studies were of a cross-sectional design, and as such, no statements can be made about causal relationships. Indeed, poor nutrition or malnutrition might contribute to frailty, or conversely, frailty might contribute to poor nutrition or malnutrition. Additionally, it is possible that some other mediating factors, such as poor dentition and swallowing problems, reduced smell and taste, or a deteriorated functional capacity that was associated with the need for feeding assistance, might have contributed to the relationship between both variables. Our review has limitations resulting from the search terms and years included. It would be really interesting, in future reviews on the topic, to include articles with malnutrition or frailty as secondary outcomes and more specific terms (protein, carbohydrate, fat, vitamin D, diet quality, dietary pattern, antioxidant, MNA, fruit or vegetable), with no limit of search years. An additional limitation is that only two studies were performed with institutionalized older adults, while seventeen were performed in community-dwelling populations, which limits significant comparisons among settings. Finally, the heterogeneity of the outcome measurements could have limited the strength of the conclusions. Nevertheless, there were also strengths that deserve to be highlighted. The main strength is that this review gives a beneficial outlook on how nutrition is linked to frailty in different elderly populations throughout the world. Thus, the results allow cross-country comparisons. In addition, we required strict criteria to define frailty, nutrition, and malnutrition in older people, and we presented a well-defined question and explicit inclusion criteria.

Establishing an optimal nutrition-based plan for the aging population should be of concern for governments for the judicious allocation of resources and for policy makers who want to add life to years and not years to life [46].

## Conclusion

This systematic review analyzes recent evidence that nutrition or nutritional intake is associated with frailty in older adults. However, a straightforward conclusion about the efficacy of nutrition on frailty cannot be established due mainly to the cross-sectional design of many of the included studies. In summary, more prospective cohort studies in older adults are needed to further understand the potential role of nutrition in the prevention, postponement, or the reversal of frailty syndrome.

## Additional files

Additional file 1: Quality assessment of the included papers (*n* = 19). (DOCX 22 kb)

Additional file 2: Reasons for the exclusion of full-text articles based on the application of inclusion criteria (*n* = 40). (DOCX 21 kb)

## Abbreviations

BDHQ: Brief-type Diet History Questionnaire
CI: Confidence interval
DHQ: Diet history questionnaire
DQI-I: Diet Quality Index-International
DQI-R: Diet Quality Index Revised
EPIC: European Prospective Investigation into Cancer and Nutrition
FFQ: Food Frequency Questionnaire
FRAIL: Fatigue, Resistance, Ambulation, Illness, Low Weight
FRALLE: Frailty Lleida
FRAP: Ferric reducing ability of plasma
InCHIANTI: Invecchiare in Chianti, ageing in the Chianti area
MDS: Mediterranean-Diet Score
MMA: Methylmalonic acid
MNA: Mini Nutritional Assessment
MNA-SF: Mini nutritional assessment short form
MrOS: Osteoporotic Fractures in Men
OR: Odds ratio
ORAC: Oxygen Radical Absorbance Capacity
RIA: Radioimmunoassay
SOF: Study of Osteoporotic Fractures
TAC: Total antioxidant capacity
TDR: Total dietary resveratrol
TEAC: Trolox equivalent antioxidant capacity
tHcy: Homocysteine
TRAP: Total radical-trapping antioxidant parameters
TUR: Total urinary resveratrol
WHAS: Women’s Health and Aging Study
