# Supplementary material for: Nutritional determinants of frailty in older adults: A systematic review
Source: BMC Geriatr. 2017 May 15;17:108. doi: 10.1186/s12877-017-0496-2 (PMC5433026; doi:10.1186/s12877-017-0496-2)
Supplement: Supplementary file 1 — Quality assessment of the included papers (n = 19). (DOCX 22 kb) [file 12877_2017_496_MOESM1_ESM.docx]

**Additional file 1. Quality assessment of the included papers (n=19)**

| **Included paper** | **Abstract** | **Rationale** | **Objectives-PICOS** | **Eligibility criteria** | **Setting** | **Study size** | **Definition of PICOS** | **Follow-up period** | **Risk of bias** | **Simple summary data** | **Effect estimates** | **Confidence intervals** | **Summary of evidence** | **Limitations** | **Conclusions** | **Funding** |
| --- | --- | --- | --- | --- | --- | --- | --- | --- | --- | --- | --- | --- | --- | --- | --- | --- |
| Bartali et al., 2006 [9] | + | + | + | + | + | + | + | - | - | +/- | - | + | + | + | + | + |
| Bollwein et al., 2013a [27] | + | + | +/- | + | + | + | + | - | - | + | - | + | + | + | + | + |
| Bollwein et al., 2013b [28] | + | + | + | + | + | + | + | - | - | + | - | + | + | + | + | + |
| Bollwein et al., 2013c [29] | + | + | + | + | +/- | + | + | - | - | + | - | - | + | + | + | + |
| Boulos et al., 2016 [33] | + | + | + | - | +/- | + | + | - | +/- | + | - | + | + | + | + | + |
| Chan R et al., 2015 [34] | + | +/- | + | +/- | + | + | + | + | - | + | - | + | + | + | + | + |
| Chang, 2017 [11] | + | + | + | + | +/- | + | + | - | - | + | - | + | + | + | + | + |
| Chang & Lin, 2016 [35] | + | + | + | + | +/- | + | +/- | - | +/- | + | +/- | - | + | + | + | + |
| El Zoghbi et al., 2014 [36] | + | +/- | + | + | + | + | + | - | +/- | + | - | + | + | + | + | + |
| Eyigor et al., 2015 [26] | + | + | + | +/- | +/- | +/- | + | - | - | + | - | + | + | + | + | - |
| Jürschik et al., 2014 [30] | + | +/- | + | +/- | +/- | +/- | + | +/- | - | + | - | - | + | + | + | + |
| Kobayashi et al., 2013 [37] | + | + | + | + | + | + | + | - | +/- | + | - | + | + | + | + | + |
| Kobayashi et al., 2014 [38] | + | + | + | + | + | + | + | - | - | + | - | + | + | + | + | + |
| Matteini et al., 2008 [39] | + | + | + | + | +/- | + | + | - | - | + | - | + | + | + | + | + |
| Michelon et al., 2006 [17] | + | +/- | + | + | + | + | + | - | +/- | + | - | + | + | + | + | + |
| Rabassa et al, 2015 [31] | + | + | + | + | + | + | + | + | +/- | + | - | + | + | + | + | + |
| Rahi et al., 2016 [32] | + | + | + | + | + | + | + | - | - | + | - | + | + | + | + | + |
| Semba et al., 2006 [10] | + | + | + | + | + | + | + | + | +/- | + | - | + | + | + | + | + |
| Shikany et al., 2014 [40] | + | +/- | + | + | + | + | + | + | +/- | + | - | + | + | + | + | + |

Abstract = Does the abstract provide a structured summary?; Rationale = Is the rationale for the study described in the context of what is already known?; Objectives = Are objectives providing an explicit statement of questions with reference to participants, interventions, comparisons, outcomes, and study design-PICOS?; Eligibility criteria = Are the eligibility criteria, sources, and methods of selection of participants clearly described?; Setting= Are the setting, locations, relevant dates, exposure, follow−up and data collection process clearly described?; Study size = Is the study size included?; Definition of PICOS = Is there reference to participants, interventions, comparisons, outcomes, and study design- PICOS?; Follow-up period = Is the follow-up period described?; Risk of bias = Is the assessment or data on risk of bias specified?; Simple summary data: Is a simple summary data presented?; Effect estimates = Are results including measures of consistency- effect estimates?; ?; Confidence intervals = Are results including confidence intervals?; Summary of evidence = Are the main findings including the strength of evidence for each main outcome and considering their relevance to key groups?; Limitations = Are limitations at study and outcome level discussed?; Conclusions = Are conclusions providing a general interpretation of the results in the context of other evidence, and implications for future research?; Funding = Are sources of funding described?
