# Supplementary material for: Nutritional determinants of frailty in older adults: A systematic review
Source: BMC Geriatr. 2017 May 15;17:108. doi: 10.1186/s12877-017-0496-2 (PMC5433026; doi:10.1186/s12877-017-0496-2)
Supplement: Supplementary file 2 — Reasons for the exclusion of full-text articles based on the application of inclusion criteria (n = 40). (DOCX 21 kb) [file 12877_2017_496_MOESM2_ESM.docx]

**Additional file 2. Reasons for the exclusion of full-text articles based on the application of inclusion criteria (n=40)**

| **Citation** | **Reason for exclusion** |
| --- | --- |
| Abellan Van Kan G, Vellas B. Is the Mini Nutritional Assessment an appropriate tool to assess frailty in older adults? J Nutr Health Aging. 2011;15(3):159-161. | Not designed to study the relationship between nutritional status and frailty |
| Akın S, Mazıcıoglu MM, Mucuk S, Gocer S, Deniz Şafak E, Arguvanlı S, et al. The prevalence of frailty and related factors in community-dwelling Turkish elderly according to modified Fried Frailty Index and FRAIL scales. Aging Clin Exp Res. 2015;27(5):703-709. | Not designed to study the relationship between nutritional status and frailty |
| Ballard J, Mooney M, Dempsey O. Prevalence of frailty-related risk factors in older adults seen by community nurses. J Adv Nurs. 2013;69(3):675-684. | Unclear criteria of frail elderly people |
| Booth J, Alex L, Francis M, Tolson D. Implementing a best practice statement in nutrition for frail older people: Part 1. Nurs Older People. 2005;16(10):26-28. | Not designed to study the relationship between nutritional status and frailty |
| Booth J, Leadbetter A, Francis M, Tolson D. Implementing a best practice statement in nutrition for frail older people: Part 2. Nurs Older People. 2005;17(1):22-24. | Not designed to study the relationship between nutritional status and frailty |
| Bradsawi M, Shahar S, Zahara AM, Nor Fadilah R, Singh DK. Efficacy of L-carnitine supplementation on frailty status and its biomarkers, nutritional status, and physical and cognitive function among prefrail older adults: a double-blind, randomized, placebo-controlled clinical trial. Clin Interv Aging. 2016;11:1675-1686. | Included persons younger than 65 |
| Carriere I, Dupuy AM, Lacroux A, Cristol JP, Delcourt C, Pathologies Oculaires Liées à l'Age Study Group. Biomarkers of inflammation and malnutrition associated with early death in healthy elderly people. J Am Geriatr Soc. 2008;56(5):840-846. | Included persons younger than 65 |
| Chen CY, Wu SC, Chen LJ, Lue BH. The prevalence of subjective frailty and factors associated with frailty in Taiwan. Arch Gerontol Geriatr. 2010;50 Suppl 1:S43-S47. | Not designed to study the relationship between nutritional status and frailty |
| Ernsth Bravell M, Westerlind B, Midlöv P, Östgren CJ, Borgquist L, Lannering C, et al. How to assess frailty and the need for care? Report from the Study of Health and Drugs in the Elderly (SHADES) in community dwellings in Sweden. Arch Gerontol Geriatr. 2011;53(1):40-45. | Not designed to study the relationship between nutritional status and frailty |
| García-Esquinas E, Rahi B, Peres K, Colpo M, Dartigues JF, Bandinelli S, et al. Consumption of fruit and vegetables and risk of frailty: a dose-response analysis of 3 prospective cohorts of community-dwelling older adults. Am J Clin Nutr. 2016;104(1):132-142. | Included persons younger than 65 |
| Gaskill D, Isenring EA, Black LJ, Hassall S, Bauer JD. Maintaining nutrition in aged care residents with a train-the-trainer intervention and Nutrition Coordinator. J Nutr Health Aging. 2009;13(10):913-917. | Not designed to study the relationship between nutritional status and frailty |
| Gurina NA, Frolova EV, Degryse JM. A roadmap of aging in Russia: the prevalence of frailty in community-dwelling older adults in the St. Petersburg district--the "Crystal" study. J Am Geriatr Soc. 2011;59(6):980-988. | Nutrition was not a primary outcome |
| Hubbard RE, O'Mahony MS, Calver BL, Woodhouse KW. Nutrition, inflammation, and leptin levels in aging and frailty. J Am Geriatr Soc. 2008;56(2):279-284. | Not designed to study the relationship between nutritional status and frailty |
| Izawa S, Enoki H, Hasegawa J, Hirose T, Kuzuya M. Factors associated with deterioration of mini nutritional assessment-short form status of nursing home residents during a 2-year period. J Nutr Health Aging. 2014;18(4):372-377. | Unclear criteria of frail elderly people |
| Izawa S, Kuzuya M, Okada K, Enoki H, Koike T, Kanda S, et al. The nutritional status of frail elderly with care needs according to the mini-nutritional assessment. Clin Nutr. 2006;25(6):962-967. | Unclear criteria of frail elderly people |
| Johnson CS, Begum MN. Adequacy of nutrient intake among elderly persons receiving home care. J Nutr Elder. 2008;27(1-2):65-82. | Unclear criteria of frail elderly people |
| Jürschik Giménez P, Escobar Bravo MÁ, Nuin Orrio C, Botigué Satorra T. Criterios de fragilidad del adulto mayor. Estudio piloto [Frailty criteria in the elderly: a pilot study]. Aten Primaria. 2011;43(4):190-196. | Not designed to study the relationship between nutritional status and frailty |
| Jyväkorpi SK, Pitkälä KH, Puranen TM, Björkman MP, Kautiainen H, Strandberg TE, et al. Low protein and micronutrient intakes in heterogeneous older population samples. Arch Gerontol Geriatr. 2015;61(3):464-471. | Included persons younger than 65 |
| Kamo T, Nishida Y. Direct and indirect effects of nutritional status, physical function and cognitive function on activities of daily living in Japanese older adults requiring long-term care. Geriatr Gerontol Int. 2014;14(4):799-805. | Not designed to study the relationship between nutritional status and frailty |
| Li CM, Chen CY, Li CY, Wang WD, Wu SC. The effectiveness of a comprehensive geriatric assessment intervention program for frailty in community-dwelling older people: a randomized, controlled trial. Arch Gerontol Geriatr. 2010;50 Suppl 1:S39-S42. | Not designed to study the relationship between nutritional status and frailty |
| Liu LK, Lee WJ, Chen LY, Hwang AC, Lin MH, Peng LN, Chen LK. Association between Frailty, Osteoporosis, Falls and Hip Fractures among Community-Dwelling People Aged 50 Years and Older in Taiwan: Results from I-Lan Longitudinal Aging Study. PLoS One. 2015;10(9):e0136968. | Included persons younger than 65 |
| Luger E, Dorner TE, Haider S, Kapan A, Lackinger C, Schindler K4. Effects of a Home-Based and Volunteer-Administered Physical Training, Nutritional, and Social Support Program on Malnutrition and Frailty in Older Persons: A Randomized Controlled Trial. J Am Med Dir Assoc. 2016;17(7):671.e9-671.e16. | Included intervention program |
| Lluis Ramos GMC. Frailty and risk associations in older adults from an urban community [Fragilidad y asociaciones de riesgo en adultos mayores de una comunidad urbana]. Rev Cub Med Mil. 2013;42(3) | Included persons younger than 65 |
| Martínez-Reig M, Gómez-Arnedo L, Alfonso-Silguero SA, Juncos-Martínez G, Romero L, Abizanda P. Nutritional risk, nutritional status and incident disability in older adults. The FRADEA study. J Nutr Health Aging. 2014;18(3):270-276. | Not designed to study the relationship between nutritional status and frailty |
| Mocchegiani E, Malavolta M, Lattanzio F, Piacenza F, Basso A, Abbatecola AM, et al. Cu to Zn ratio, physical function, disability, and mortality risk in older elderly (ilSIRENTE study). Age. 2012;34(3):539-552. | Did not include a sample of frail elders |
| Odlund Olin A, Koochek A, Cederholm T, Ljungqvist O. Minimal effect on energy intake by additional evening meal for frail elderly service flat residents--a pilot study. J Nutr Health Aging. 2008;12(5):295-301. | Unclear criteria of frail elderly people |
| Perna S, Francis MD, Bologna C, Moncaglieri F, Riva A, Morazzoni P, et al. Performance of Edmonton Frail Scale on frailty assessment: its association with multi-dimensional geriatric conditions assessed with specific screening tools. BMC Geriatrics. 2017;17:2. | Not designed to study the relationship between nutritional status and frailty |
| Rydwik E, Lammes E, Frändin K, Akner G. Effects of a physical and nutritional intervention program for frail elderly people over age 75. A randomized controlled pilot treatment trial. Aging Clin Exp Res. 2008;20(2):159-170. | Unclear criteria of frail elderly people |
| Sánchez-García S, Sánchez-Arenas R, García-Peña C, Rosas-Carrasco O, Avila-Funes JA, Ruiz-Arregui L, et al. Frailty among community-dwelling elderly Mexican people: prevalence and association with sociodemographic characteristics, health state and the use of health services. Geriatr Gerontol Int. 2014;14(2):395-402. | Included persons younger than 65 |
| Serra Prat M, Fernández X, Ribó L, Palomera E, Papiol M, Serra P. Pérdida de apetito en ancianos no institucionalizados y su relación con la capacidad functional [Loss of appetite in elderly people in the community and its relationship with functional capacity]. Med Clin (Barc). 2008;130(14):531-533. | Unclear criteria of frail elderly people |
| Serra-Prat M, Palomera E, Clave P, Puig-Domingo M. Effect of age and frailty on ghrelin and cholecystokinin responses to a meal test. Am J Clin Nutr. 2009;89(5):1410-1417. | Patients were on a specific diet |
| Serra-Prat M, Papiol M, Vico J, Palomera E, Sist X, Cabré M. Factors associated with frailty in community-dwelling elderly population. A cross-sectional study. Eur Geriatr Med. 2016;7:531-537. | Nutrition was not a primary outcome |
| Serra-Prat M, Sist X, Domenich R, Jurado L, Saiz A, Roces A, et al. Effectiveness of an intervention to prevent frailty in pre-frail community-dwelling older people consulting in primary care: a randomized controlled trial. Age Ageing. 2017, Jan 6. doi: 10.1093/ageing/afw242. | Included intervention program |
| Serra-Prat M, Sist X, Saiz A, Jurado L, Domenich R, Roces A, et al. Clinical and Functional Characterization of Pre-frailty among Elderly Patients Consulting Primary Care Centres. J Nutr Health Aging. 2016;20(6):653-658. | Not designed to study the relationship between nutritional status and frailty |
| Sewo Sampaio PY, Sampaio RA, Coelho Júnior HJ, Teixeira LF, Tessutti VD, Uchida MC, et al. Differences in lifestyle, physical performance and quality of life between frail and robust Brazilian community-dwelling elderly women. Geriatr Gerontol Int. 2016;16(7):829-835. | Included persons younger than 65 |
| Sirola J, Pitkala KH, Tilvis RS, Miettinen TA, Strandberg TE. Definition of frailty in older men according to questionnaire data (RAND-36/SF-36): The Helsinki Businessmen Study. J Nutr Health Aging. 2011;15(9):783-787. | Not designed to study the relationship between nutritional status and frailty |
| Slee A, Birch D, Stokoe D. A comparison of the malnutrition screening tools, MUST, MNA and bioelectrical impedance assessment in frail older hospital patients. Clin Nutr. 2015;34(2):296-301. | Unclear criteria of frail elderly people |
| Sourial N, Wolfson C, Bergman H, Zhu B, Karunananthan S, Quail J, Fletcher J, et al. A correspondence analysis revealed frailty deficits aggregate and are multidimensional. J Clin Epidemiol. 2010;63(6):647-654. | Nutrition was not a primary outcome |
| Takeuchi K, Aida J, Ito K, Furuta M, Yamashita Y, Osaka K. Nutritional status and dysphagia risk among community-dwelling frail older adults. J Nutr Health Aging. 2014;18:352-357. | Unclear criteria of frail elderly people |
| Woo J, Yu R, Wong M, Yeung F, Wong M, Lum C. Frailty Screening in the Community Using the FRAIL Scale. J Am Med Dir Assoc. 2015;16(5):412-419. | Nutrition was not a primary outcome |
